# Identification of preschoolers with special educational needs: comparing the discriminative validity of the Strengths and Difficulties Questionnaires (SDQ) and the Achenbach System of Empirically Based Assessment (ASEBA) across different informants in Hong Kong

**DOI:** 10.3389/fpsyg.2025.1623690

**Published:** 2025-08-29

**Authors:** Terry Tin-Yau Wong, Jacqueline Wai-Yan Tang, Pokky Poi-Ki Choi, Terence Siu-Chung Leung

**Affiliations:** ^1^Department of Psychology, University of Hong Kong, Pokfulam, Hong Kong SAR, China; ^2^Caritas Rehabilitation Service, Hong Kong, Hong Kong SAR, China

**Keywords:** SDQ, ASEBA, preschool, behavioral and emotional problems, mental health

## Abstract

**Background:**

Early behavioral and emotional problems are associated with poor developmental outcomes. It is thus important to identify preschoolers with behavioral and emotional problems so that effective interventions can be provided for them early. The current study aimed to compare the screening efficiency of the parent and teacher versions of the Strengths and Difficulties Questionnaire (SDQ) and the Achenbach System of Empirically Based Assessment (ASEBA) in identifying children with early behavioral and emotional problems.

**Method:**

A community sample (*n* = 312) aged 3 to 5, as well as a clinical sample (*n* = 79) of the same age, were recruited. Parents and teachers of these participants completed the relevant forms of SDQ as well as the Child Behavior Checklist for Ages 1.5–5 (CBCL/1½-5)/Caregiver-Teacher Report Form (C-TRF).

**Results:**

Both instruments yielded satisfactory internal consistency and test–retest reliabilities. Teachers’ reports were more accurate in terms of differentiating the clinical sample from the community sample, and the SDQ-T yielded more consistent discriminative validity across different ages.

**Discussion:**

Psychologists, psychiatrists and allied healthcare professionals are recommended to use teachers’ report, or the SDQ-T in particular, to identify preschoolers who may require further assessment for their behavioral and emotional issues.

## 1 Introduction

Behavioral and emotional problems observed during early childhood can have a huge impact on children’s development. For instance, young children who were rated as having greater behavioral and emotional problems were shown to have worse academic performance (Washbrook et al., 2013) and greater chances of being diagnosed with mental disorders (Nielsen et al., 2019) during their adolescence. Cross-cultural research findings also suggest that childhood attention and behavior problems were associated with a range of outcomes such as less earning, lower educational attainment, poorer mental and physical health in adulthood (Koepp et al., 2023). These longitudinal research findings shed light on the importance of early screening and intervention of childhood behavioral problems.

### 1.1 Screening tools for identifying behavioral and emotional problems in early childhood

The use of screening questionnaires allows clinicians to effectively and efficiently identify young children who may warrant special attention. The Strengths and Difficulties Questionnaires (SDQ) and the Achenbach System of Empirically Based Assessment (ASEBA) are the most commonly used questionnaires that help identify children with behavioral and emotional problems (Mulraney et al., 2022). The SDQ was initially developed by Goodman (1997) as a brief behavioral screening questionnaire covering children’s and adolescents’ behaviors, emotions, and relationships. This 25-item questionnaire, which can be rated by both parents and teachers, assesses children’s behavioral and emotional difficulties and strengths along five domains, namely emotional symptoms, conduct problems, hyperactivity/ inattention, peer relationship problems, and prosocial behaviors, with equal number of items within each domain. The domain scores, except for the prosocial behavior scores, can then be combined to form a total difficulties score.

The ASEBA system was initially developed to identify syndromes of co-occurring problems seen among disturbed children and adolescents (Achenbach and Rescorla, 2004). The three sets of questionnaires in the ASEBA system, namely the Child Behavior Checklist (CBCL), Teacher Report Form (TRF), and the Youth Self-Report (YSR) are commonly used by clinicians as screeners for identify children and adolescents with behavioral and emotional issues. As the current study focused on preschoolers, the Child Behavior Checklist for Ages 1½-5 (CBCL/1½-5) as well as the Caregiver-Teacher Report form for Ages 1½-5 (C-TRF) were used in this study. Both questionnaires included 100 items. These items were categorized into six domains in the C-TRF (i.e., emotionally reactive, anxious/depressed, somatic complaints, withdrawn, attention problems, and aggressive behaviors), which were further summarized as internalizing problems (covering the first four domains), externalizing problems (covering the last two domains), and total problems (covering all six domains). The CBCL/1½-5 also included a sleep problem domain, which was not included in either the internalizing or externalizing problems scores but included in the total problem scores.

### 1.2 The screening efficiency of SDQ versus ASEBA

To fully utilize these screeners for identifying preschoolers who need further assessment, clinicians need to consider the statistical properties as well as the practical efficiency of these screeners.

First of all, the comparable subscales of the ASEBA and SDQ appeared to be measuring highly similar constructs, as their correlations range from 0.58 to 0.75. These strong correlations indicate substantial convergence between the measures despite their different lengths. This demonstrates that while the instruments differ in format and length, they measure similar underlying constructs (Mansolf et al., 2022).

Second, in terms of internal consistency, ASEBA has demonstrated stronger internal consistency across its scales (0.76–0.96) compared to the SDQ, which shows excellent consistency for the Total Problems scale (0.81) but only poor to fair consistency for its subscales (0.31–0.73). This discrepancy in reliability reflects the trade-off between comprehensiveness and brevity (Dang et al., 2017).

More importantly, the relative discriminative validity of SDQ versus ASEBA were examined in different studies, but their conclusions did not always align. The findings from Dang et al. (2017), for instance, illustrated that the CBCL did a better job in terms of differentiating a group of inpatients and outpatients aged 6 to 16 from a community-based sample of the same age range. The findings from Klasen et al. (2000), however, suggested that despite the brevity of the SDQ, it outperformed the CBCL in differentiating between the community sample and the clinical sample. In a systematic review, Warnick et al. (2008) showed that the CBCL and the SDQ showed similar screening efficiencies as the likelihood ratio estimates, or the likelihood of detecting psychiatric disorders, of the two instruments did not differ significantly (CBCL likelihood ratio estimates = 4.87, SDQ likelihood ratio estimates = 5.02). However, only three studies on SDQ were included in Warnick et al.’s (2008) systematic review. Given the contrasting findings, there is no clear conclusion concerning which instrument yielded higher discriminative validity in terms of identifying children with behavioral and emotional problems.

The length difference between the two instruments (ASEBA: 100–119 items; SDQ: 25 items) represents a key consideration for clinical practice, with the SDQ’s brevity offering practical advantages. Mansolf et al. (2022), for example, asserted that when broader domain scores are used rather than specific syndrome measures, the reliability differences between the instruments become less pronounced, and the brevity of the instrument may become a greater concern in the instrument selection process.

### 1.3 The relationship between informants and screening efficiency

Another issue that clinicians should be concerned about is whether parent or teacher ratings serve as better indicators of the child’s behavioral and emotional status. Parents and teachers observe the same child in different settings, and this may explain why their ratings of the behaviors of the same child do not always agree. Cross-informant correlations for behavioral and emotional problems typically achieve only moderate agreement (Rescorla et al., 2014). Research by Kersten et al. (2016) demonstrates this limited consistency, with weighted average correlation coefficients falling between just 0.25 and 0.45, indicating only weak to moderate agreement across different informants.

This raised the issue of whether parent or the teacher ratings better differentiate the clinical samples from the community samples. The literature suggested that parent ratings are generally more indicative of a child’s clinical status (Kersten et al., 2016; Mulraney et al., 2022; Stone et al., 2010). For instance, Mulraney et al. (2022) had compared the screening accuracies of various screening tools for attention-deficit/hyperactivity disorder and concluded that parent ratings, compared to teacher ratings, are generally more accurate in terms of differentiating children with ADHD from the community sample. These authors attributed this to the issue of shared method variance, as the diagnostic interview of ADHD is usually done with parents, not teachers. Meanwhile, it can also be argued that teachers should have received more training concerning children’s development in general, and they should have more opportunities to compare a particular child with his/her same-age peers. Both factors should have prepared the teachers in spotting the abnormality among children. The findings from Du et al. (2008), which were based on a sample that is more culturally similar to the current sample, provided support to this argument by showing that teachers’ ratings did a much better job in differentiating an ADHD sample from the community sample. The conflicting findings and arguments have prevented us from concluding whether parent or teacher ratings are generally better than teacher ratings in terms of indicating a child’s clinical status, or the parent advantage is culture specific.

### 1.4 The current study

Although the issues of how the instrument and the informant affect the discriminative validity of the screening process have received some attention in the literature, the findings are not conclusive for the following reasons. First, a cross-sectional study involving seven different countries suggested that there are huge cross-national variations in these screeners (Goodman et al., 2012). Such cross-national differences suggest that the absolute and relative level of discriminative validity of the instruments may vary across cultures, which therefore justify the needs for culture-specific studies. Second, among the existing validation studies of the two instruments, the focus was usually placed on the school-age population (Stone et al., 2010; Warnick et al., 2008). Less is known about the discriminative validity of both instruments in the preschool population. The preschool environment is much less structured compared to the school environment, and the findings from school-age populations may not always generalize to the preschool settings.

Given the inconsistent findings concerning the relative discriminative validity of the SDQ and the ASEBA system, which was further compounded by the potential moderating roles of informant and culture as well as the limited investigation of the topic among the preschool population, the current study was conducted to compare the ability of the SDQ and the ASEBA system in differentiating a clinical preschool sample from a community preschool sample in Hong Kong. We also aimed to compare the discriminative validity of parents’ versus teachers’ ratings.

## 2 Method

### 2.1 Participants

#### 2.1.1 Community sample

The community sample consists of a total of 312 preschoolers aged 3.0 to 5.11 from 16 preschools and kindergartens in Hong Kong. The participants were recruited through a stratified random sampling procedure, which resulted in a community sample that is representative of the preschool population in Hong Kong in terms of geographical locations and household income by district (see Table 1). The community sample is evenly distributed in terms of age and gender (see Table 2).

**Table 1:** The geographic location of the community sample in relation to the preschool population in Hong Kong.

Geographic location	Sample Size *n* (%)	Preschool population in Hong Kong *n* (%)	Household income by district		
			High	Medium	Low
Hong Kong Island	51 (16%)	26,908 (16%)	51	/	/
Kowloon	99 (32%)	54,561 (33%)	/	54	45
The New Territories	162 (52%)	83,466 (51%)	55	56	51
Total	312 (100%)	164,935 (100%)	106	110	96

**Table 2:** The number of participants by age and gender.

Age	Community sample			Clinical sample		
	Male	Female	Total	Male	Female	Total
3;0–3;11	50	45	95	15	10	25
4;0–4;11	57	56	113	13	10	23
5;0–5;11	55	49	104	21	10	31
Total	162	150	312	49	30	79

Within the community sample, a convenient sub-sample of 55 participants was invited for retesting, and their parents and teachers complete the questionnaires again within 1–4 weeks after the initial completion of the questionnaire.

#### 2.1.2 Clinical sample

A total of 79 preschoolers from kindergartens/kindergarten-cum-child care centres participating in the On-site Preschool Rehabilitation Services in Hong Kong was recruited to comprise the clinical sample. These kindergartens/ kindergarten-cum-child care centres provide preschool rehabilitation services to children with special needs, and only students with diagnoses (e.g., global developmental delay, autism spectrum disorder, attention deficit/hyperactivity disorder, etc.) by pediatricians or psychologists are entitled to these services. The age and gender distributions of the clinical sample are listed in Table 2.

### 2.2 Measures

#### 2.2.1 SDQ

The SDQ is a brief screening questionnaire developed by Goodman (1997) to identify children with mental health issues and special needs. Informants (i.e., parents and teachers) were asked to rate the child on these 25 items using a 3-point Likert scale. Items can be summarized into 5 scales scores as well as a total difficulties score. The Chinese versions of the SDQs, translated by the Chinese University of Hong Kong, were downloaded directly from the SDQ official website (https://www.sdqinfo.org/) and used in the current study. Two versions of the SDQs were used: the 2–4 year olds version was used for children aged 3; while the 4–17 year olds version was used for children aged 4 and 5.

#### 2.2.2 CBCL for ages 1.5–5 and caregiver-teacher report form (CBCL/1½-5 and C-TRF)

The Chinese version of CBCL/1½-5 and C-TRF (Leung et al., 2006), two sets of questionnaires in the Achenbach System of Empirically Based Assessment (Achenbach and Rescorla, 2004), were completed by parents and teachers of the participants. CBCL/1½-5 and C-TRF are sets of comprehensive questionnaires tapping various areas of psychopathologies and mental health issues. There are over 100 items within each questionnaire, each of them is rated on a 3-point Likert scale. The scores can be grouped into several syndrome scores as well as three summary scores: internalizing problems, externalizing problems, and total problems.

### 2.3 Procedures

Ethics approval of the current project was obtained from the first author’s affiliated university. Participating schools and centres helped distribute and collect parental consent from participants’ parents. Only participants with parental consent were included in the study. For each participant, two sets of questionnaires were given to their teachers (SDQ-T and C-TRF), and two sets of questionnaires were given to their parents (SDQ-P and CBCL/1½-5). Questionnaires were distributed in the second semester so that the teachers should have known the children for at least 6 months.

### 2.4 Analyses

First, descriptive statistics were reported. The main effects of age and gender on the parent and teacher ratings were examined using a two-way MANOVA. Internal consistency (Cronbach’s *α*) and test–retest reliability (Intraclass correlations) were reported as reliability indicators. Correlations were computed to examine the inter-rater reliabilities and the convergent validity between SDQ and the ASEBA system.

Next, the discriminative validity of the SDQs and the ASEBA system was assessed by comparing the ratings between the clinical and the community samples using three one-way MANCOVAs, one for each age group, controlling for the effect of gender. Receiver Operating Characteristics (ROC) analyses were also conducted, and the AUC of the domain scores were computed. AUCs of greater than 0.90, between 0.80 to 0.90, and between 0.70 to 0.80, and smaller than 0.70 were considered as excellent, good, fair, and poor, respectively, (Youngstrom, 2014). Lastly, the optimal cutoff values were reported to facilitate clinicians in effectively screening children with emotional and behavioral difficulties.

## 3 Results

Missing data appeared in 14% of the community sample and 19% of the clinical sample. Participants with missing data did not seem to differ from participants with complete data in terms of age, parental education, and family income, |*t|*s < 1.6, *p*s > 0.15, but there are more girls among the participants with missing data, *t* = −2.519, *p* = 0.012. Participants with missing data were excluded from the following analyses.

### 3.1 Descriptive statistics

The total difficulties scores of SDQ-P and SDQ-T, as well as the total problem scores of CBCL/1½-5 and C-TRF, of the community sample participants were summarized in Table 3. The effects of age and gender were examined using two-way ANOVAs. The effects of age and gender were only observed in teacher-reported rating scales. Girls scored lower than boys in both SDQ-T [*F*(1,262) = 14.04, *p* < 0.001, *η_p_^2^* = 0.051] and C-TRF [*F* (1,262) = 6.02, *p* = 0.015, *η_p_^2^* = 0.022]. A significant main effect of age was observed in SDQ-T only [*F* (2,262) = 3.99, *p* = 0.02, *η_p_^2^* = 0.030]. Post-hoc analysis with Bonferroni adjustment revealed a significantly lower total problem difficulties score in SDQ-T in 5-year-olds (*M* = 8.92, *SD* = 5.23) than 4-year-olds (*M* = 11.10, *SD* = 6.27; *p* = 0.029). None of the age × gender interaction was significant.

**Table 3:** Descriptive statistics and reliability information of the summary scores of SDQ-P, SDQ-T, CBCL/1½-5, and C-TRF.

Screening tool	α (A3/A4-5)	ICC (A3/A4-5)	Total (*n* = 268)	Male – 3 (*n* = 45)	Male – 4 (*n* = 49)	Male – 5 (*n* = 47)	Female – 3 (*n* = 41)	Female – 4 (*n* = 47)	Female – 5 (*n* = 39)	Effect size of age (η_p_^2^)	Effect size of gender (η_p_^2^)	Effect size of age x gender interaction (η_p_^2^)
SDQ-P	0.710/0.836	0.837/0.820	12.51 (5.58)	13.42 (4.41)	12.98 (4.96)	12.87 (6.32)	12.85 (4.88)	11.91 (6.91)	10.82 (5.36)	0.009	0.012	0.003
SDQ-T	0.825/0.834	0.863/0.670	10.28 (5.83)	11.91 (5.49)	12.65 (5.90)	9.89 (5.34)	9.44 (5.75)	9.49 (6.28)	7.74 (4.92)	0.030*	0.051***	0.001
CBCL/1½-5	0.959/0.971	0.878/0.888	35.08 (25.53)	35.40 (23.89)	35.61 (21.47)	35.55 (29.88)	37.15 (22.06)	35.60 (28.93)	30.67 (26.53)	0.003	0.000	0.003
C-TRF	0.962/0.962	0.837/0.876	20.94 (20.63)	24.31 (21.97)	25.88 (19.65)	20.98 (21.26)	18.22 (19.42)	21.32 (23.78)	13.21 (14.16)	0.017	0.022*	0.001

*p < 0.05, ***p < 0.001.

### 3.2 Reliability

#### 3.2.1 Internal consistency

The internal consistencies of SDQ-P and SDQ-T, as well as those of CBCL/1½-5 and C-TRF, were shown in Table 3. All scaled yielded satisfactory internal consistency, with Cronbach’s αs being greater than 0.7. However, the internal consistencies of CBCL/1½-5 and C-TRF, which were above 0.95, were higher than those of SDQ-P and SDQ-T, which fell between the range of 0.70 to 0.85.

#### 3.2.2 Test–retest reliability

Test–retest reliabilities, calculated using the intra-class correlations (ICC), were shown in Table 3. With the exception of SDQ-T among children aged 4–5 (ICC = 0.67), all other test–retest reliabilities were satisfactory, with ICCs being greater than 0.80.

### 3.3 Correlations

Correlations among the total difficulties scores of SDQ-P and SDQ-T, as well as the total problem scores of CBCL/1½-5 and C-TRF, were presented in Table 4. The ratings by the same informants (i.e., SDQ-P with CBCL/1½-5, SDQ-T with C-TRF) correlated strongly with each other (*r*s > 0.62, *p*s < 0.01). The interrater reliability across informants, however, fell only in the moderate range (0.26 < *r*s < 0.36). The findings suggested a higher level of convergence across instruments than across informants.

**Table 4:** Correlations among the summary scores.

	SDQ-P	SDQ-T	CBCL/1½-5	C-TRF
SDQ-P	–	0.265*	0.626***	0.260*
SDQ-T	0.278***	–	0.196	0.814***
CBCL/1½-5	0.795***	0.195**	–	0.323**
C-TRF	0.351***	0.757***	0.358***	–

Numbers above/below the diagonal represent correlations for age 3/ age 4–5, respectively.

*p < 0.05, **p < 0.01, ***p < 0.001.

### 3.4 Validity

#### 3.4.1 Comparison of group means

The means and standard deviations of the community sample and the clinical samples on the four summary scores were summarized in Table 5. Due to the main effects of age and gender observed in some of the questionnaires, the use of different forms of SDQ for children aged 3 versus 4–5, as well as the limited number of girls in the clinical sample, it was decided to examine the group differences in three separate MANCOVAs, one for each age group, with gender serving as the covariate in these analyses. In general, the parents’ ratings were very similar for the community sample and the clinical sample, the only contrasts that were significant were observed among the 4-year-olds [SDQ-P: *F* (1,108) = 5.59, *p* = 0.020, *η_p_^2^* = 0.049; CBCL/1½-5: *F* (1,108) = 13.39, *p* < 0.001, *η_p_^2^* = 0.110]. On the other hand, large differences between the community sample and the clinical sample were observed in teachers’ ratings, with all the group differences being statistically significant with medium to large effect sizes, *F* (1,108)s > 5.9, *p*s < 0.02, *η_p_^2^* ≥ 0.05.

**Table 5:** Group differences between the community sample and the clinical sample in terms of the summary scores.

Screening tool	Age	Community sample	Clinical sample	Effect size (η_p_^2^)	AUC
SDQ-P	3	13.15 (4.62)	14.47 (4.82)	0.013	0.576
	4	12.46 (5.99)	16.73 (5.48)	0.049*	0.723
	5	11.94 (5.97)	12.27 (5.40)	0.000	0.525
SDQ-T	3	10.73 (5.72)	15.79 (5.51)	0.083**	0.750
	4	11.10 (6.27)	16.60 (3.70)	0.066**	0.790
	5	8.92 (5.23)	12.80 (5.72)	0.084**	0.720
CBCL/1½-5	3	36.23 (22.92)	40.05 (20.80)	0.007	0.569
	4	35.60 (25.27)	67.00 (34.64)	0.138***	0.778
	5	33.34 (28.35)	34.63 (26.00)	0.000	0.517
C-TRF	3	21.41 (20.90)	45.53 (26.75)	0.101***	0.759
	4	23.65 (21.77)	48.27 (26.12)	0.110***	0.771
	5	17.45 (18.69)	29.70 (28.38)	0.050*	0.650

*p < 0.05, **p < 0.01, ***p < 0.001.

#### 3.4.2 Discriminative validity

Results of ROC analyses echoed the group comparison results in general: most of the parents’ ratings, except those for children aged 4 (SDQ-P AUC = 0.723; CBCL/1½-5 AUC = 0.778), did not yield satisfactory AUC. Teachers’ ratings, however, yielded higher discriminative validity in terms of differentiating the clinical group from the community group, with five out of six of the AUCs being greater than 0.7. The findings suggested that teachers appear to be better able to differentiate typically developing children from children who may need rehabilitation services. In terms of the comparison across rating scales, SDQ-T appeared to be more consistent in differentiating the clinical sample from the community sample across the age range of 3 to 5, with its AUCs being consistently above 0.70. Given the brevity of the SDQ-T and its consistently good performance in differentiating the clinical samples from the community sample, the SDQ-T total difficulties score was recommended for identifying preschool children who may need rehabilitation services.

#### 3.4.3 Cutoff scores

Based on the score distribution of the SDQ-T total difficulties scores, we explored the sensitivities, specificities, positive predictive values, negative predictive values, and overall classification accuracies across the three age groups by varying the cutoff values (see Table 6). While the two cutoff values proposed by Goodman (1997) and Lai et al. (2010) (i.e., 90th and 85th percentiles respectively) resulted in high specificities (SP ≥ 0.80), the sensitivities were low (SE ≤ 0.47). The cutoff was adjusted downwards to a T score of approximately 54, which resulted in comparable sensitivities and specificities (Age 3: SE = 0.68, SP = 0.69; Age 4: SE = 0.80, SP = 0.73; Age 5: SE = 0.73, SP = 0.72). Given the purpose of SDQ was to screen children who may need further assessments, sensitivity was valued over specificity, and the cutoff T value of 54 was recommended.

**Table 6:** Cutoff scores for SDQ-T total difficulties score.

Age	Percentile	Raw score	T score	Sensitivity	Specificity	PPV	NPV	Overall acc.
Age 3	90th percentile	≥ 19	64	0.32	0.87	0.35	0.85	0.77
	85th percentile	≥ 16	59	0.47	0.85	0.41	0.88	0.78
	Suggested	≥ 13	54	0.68	0.69	0.33	0.91	0.69
Ages 4	90th percentile	≥ 20	64	0.33	0.90	0.33	0.90	0.82
	85th percentile	≥ 18	61	0.47	0.83	0.30	0.91	0.78
	Suggested	≥ 14	55	0.80	0.73	0.32	0.96	0.74
Age 5	90th percentile	≥ 17	65	0.20	0.90	0.40	0.76	0.72
	85th percentile	≥ 13	58	0.43	0.81	0.45	0.80	0.72
	Suggested	≥ 11	54	0.73	0.72	0.48	0.89	0.72

## 4 Discussion

The current study was conducted to examine the discriminative validity of two commonly used mental health screeners among a Chinese preschool population. A community sample that is representative of the preschool population of the city, as well as a clinical sample that was recruited from centres that provided rehabilitation services to preschool children with special needs, were recruited for the purpose. These preschoolers were rated by both their parents and their teachers on their behavioral and emotional issues. The ratings of the clinical sample were compared to those of the community sample to examine the absolute and relative discriminative validity of the SDQ-P, SDQ-T, CBCL/1½-5, and C-TRF. The SDQ-T appeared to perform most consistently in terms of differentiating the clinical sample from the community sample.

### 4.1 Reliability of the SDQs and the ASEBA system

Generally speaking, both sets of questionnaires showed adequate internal consistency and test–retest reliabilities (except for test–retest reliability of SDQ-T among 4- to 5-year-olds). Comparatively speaking, the ASEBA system (αs > 0.95) showed higher internal consistency than the SDQs (0.70 < αs < 0.85). The findings align with those from Dang et al. (2017), which was also conducted in the Asian context. The higher internal consistency in the ASEBA system, compared to that in the SDQ, could be explained by the fact that the ASEBA system contained 4 times as many items as the SDQs. Except for the lower test–retest reliability of SDQ-T among 4- to 5-year-olds, the test–retest reliability of parents and teachers’ ratings are largely similar.

The interrater reliability of both instruments, however, fell only within the moderate range (0.26 < *r*s < 0.36). This moderate level of interrater reliability in other studies as well. In a large-scale cross-scale cross-cultural study with over 27,000 participants across 21 societies, the interrater reliability of CBCL versus TRF was found to be 0.26, with a range of 0.09 to 0.49 (Rescorla et al., 2014). In a more recent meta-analysis, the mean correlation between parent and teacher ratings on preschoolers’ emotional and behavioral problems was 0.28, and the range could go from −0.41 to 0.54 (Carneiro et al., 2021). The fact that parents and teachers observe the same child in different settings may have contributed to this moderate level of interrater reliability. Because parents’ and teachers’ ratings showed only moderate correlations, it is worthwhile to further examine whether parents’ or teachers’ ratings served as better indicator of children’s behavioral and emotional issues.

### 4.2 Validity of the SDQs and the ASEBA system

The validity of the SDQ was examined through various sources. First, the overall summary scores of the two sets of questionnaires completed by the same informants were strongly correlated with each other, with a mean correlation of 0.75 (0.62 < *r*s < 0.82). The figure aligned very well with those observed in a review by Stone et al. (2010), which suggested a weighted correlation of 0.76 between SDQ total difficulties scores and CBCL total problem scores. These findings suggest that the two sets of questionnaires assess highly similar constructs. The findings confirm the convergent validity of both instruments.

The validity of the SDQs and the ASEBA system was further examined by comparing the ratings of the clinical sample with those of the community sample after controlling for the effect of gender. Similar to the findings from Warnick et al. (2008), the current findings suggested that both the SDQ and the ASEBA system appeared to perform similarly in terms of differentiating the clinical sample from the community sample. Yet, informant appeared to play a larger role in terms of determining the discriminative validity. While significant differences between the clinical sample and the community sample were consistently observed in teachers’ ratings, such significant differences were observed in parents’ ratings only among 4-year-olds. The pattern was the same for both instruments. The higher discriminative validity among teachers’ ratings, compared to that of the parents’ ratings, was also observed in another sample (Du et al., 2008). The teacher training that preschool teachers have received, together with their experience of interacting with many children of the same age in the classroom, may put teachers in better positions to differentiate problematic behaviors from typical behaviors among children. However, the higher discriminative validity of the teachers’ ratings compared to the parents’ ratings was not consistently observed in other studies (see Kersten et al., 2016, for a review). It is interesting to note that both studies that support the superiority of teacher rating in predicting children’s clinical status were conducted in the Chinese context (i.e., the current study and Du et al., 2008), and it is possible that such a teacher advantage may be culturally specific. Further studies are needed to examine whether culture plays a role in moderating the relative discriminative validity of parents’ versus teachers’ ratings.

### 4.3 Recommendations for clinicians

Among the four sets of questionnaires being examined, the total difficulties score of SDQ-T appeared to be the best index that differentiated the clinical sample from the community sample. Its discriminative validity was consistent across different ages, and it was at least as discriminative, if not more discriminative, than the relevant summary scores in the other three questionnaires. The strong discriminative power of the SDQ-T, combined with its brevity, has made it a better candidate for screening children who need special care in the preschool setting. On top of SDQ-T, clinicians should also consider other relevant information obtained from other sources (e.g., interviews, observation, parental ratings) before making a clinical decision.

While the 90th percentile and the 85th percentile had been proposed by previous researchers for identifying children who need services (Goodman, 1997; Lai et al., 2010), both cutoffs resulted low sensitivities (SEs < 0.50). As the major goal of mental health screeners was to screen out children who may need services, a lower cutoff is probably desirable in order not to miss too many children. A cutoff value of T = 54 was proposed in the current study, which resulted in sensitivities of approximately 0.70. Local preschool children who receive a T score of 54 or above (equivalent to a raw score of 13, 14, and 11 for age 3, 4, and 5 respectively) in SDQ-T are recommended to visit a psychologist or a psychiatrist for further assessments of their developmental and mental health needs.

### 4.4 Limitations and future directions

Readers should note that while the total difficulties score of SDQ-T significantly differentiated children with special needs from their typically developing peers, the discriminative validity was only in the satisfactory range. In fact, the AUC of SDQ-T observed in the current study (i.e., between 0.72 and 0.79) appeared to be slightly lower than the weight AUC of 0.83 observed in the review by Stone et al. (2010). Even after lowering the cutoff values to T = 54, which resulted in specificities of approximately 0.70, the sensitivities were still only around 0.70. This may be due to the fact that the clinical sample in the current study is comprised of students with an assortment of special needs, including some to which both questionnaires may not be sensitive to, such as global developmental delays, early signs of dyslexia, physical disabilities, speech and language pathologies, and so on. Further studies may include more specific clinical samples that the SDQ and the ASEBA system are sensitive to, that is, including only children with disorders like Attention-Deficit Hyperactivity Disorders and Autism Spectrum Disorders, to examine if the sensitivity and specificity values could be enhanced when the prediction are more specific. Furthermore, the limited number of girls in the clinical sample prevented us from separating our analyses by gender. One potential reason for this is that within the clinical sample, girls’ emotional and behavioral problems were less severe than the boys’ ones, and the parents of girls in the clinical sample may not see a need to complete the questionnaires. Future studies may include a larger female clinical sample, as well as to more specifically educate the parents of girls about child psychopathology to reduce drop-out, in order to provide more accurate diagnostic information of the instruments.

## 5 Conclusion

The current study was among the very few studies that compared the discriminative validity of the SDQs and the ASEBA system within a preschool population. Both instruments, with either parents or teachers serving as informants, showed adequate internal consistency and test–retest reliability. The internal consistency of the ASEBA system fell within the excellent range. Concerning discriminative validity, the teachers’ ratings appeared to do a better job in terms of differentiating the clinical sample from the community samples. Because of its brevity as well as its consistent performance in identifying the clinical sample across different ages, the total difficulties score of SDQ-T was therefore recommended for identifying at-risk preschool children, who may receive early interventions that may improve their academic achievement, social relationship, as well as their mental health during adolescence and adulthood.

## Data Availability

The raw data supporting the conclusions of this article will be made available by the authors, without undue reservation.
