# Correction: Identification of preschoolers with special educational needs: comparing the discriminative validity of the Strengths and Difficulties Questionnaires (SDQ) and the Achenbach System of Empirically Based Assessment (ASEBA) across different informants in Hong Kong

**DOI:** 10.3389/fpsyg.2025.1703161

**Published:** 2025-10-06

**Authors:** Terry Tin-Yau Wong, Jacqueline Wai-Yan Tang, Pokky Poi-Ki Choi, Terence Siu-Chung Leung

**Affiliations:** ^1^Department of Psychology, University of Hong Kong, Pokfulam, Hong Kong SAR, China; ^2^Caritas Rehabilitation Service, Hong Kong, Hong Kong SAR, China

**Keywords:** SDQ, ASEBA, preschool, behavioral and emotional problems, mental health

There was a mistake in Table 6 as published. The T score column was missing. The corrected Table 6 appears below.

**Table 6:** Cutoff scores for SDQ-T total difficulties score.

**Age**	**Percentile**	**Raw score**	**T score**	**Sensitivity**	**Specificity**	**PPV**	**NPV**	**Overall acc**.
3	90th percentile	≥19	64	0.32	0.87	0.35	0.85	0.77
	85th percentile	≥16	59	0.47	0.85	0.41	0.88	0.78
	Suggested	≥13	54	0.68	0.69	0.33	0.91	0.69
4	90th percentile	≥20	64	0.33	0.90	0.33	0.90	0.82
	85th percentile	≥18	61	0.47	0.83	0.30	0.91	0.78
	Suggested	≥14	55	0.80	0.73	0.32	0.96	0.74
5	90th percentile	≥17	65	0.20	0.90	0.40	0.76	0.72
	85th percentile	≥13	58	0.43	0.81	0.45	0.80	0.72
	Suggested	≥11	54	0.73	0.72	0.48	0.89	0.72

The original version of this article has been updated.

